# Traction force generation in *Plasmodium* sporozoites is modulated by a surface adhesin

**DOI:** 10.1111/jmi.70015

**Published:** 2025-07-26

**Authors:** Johanna Ripp, Dimitri Probst, Mirko Singer, Ulrich S. Schwarz, Friedrich Frischknecht

**Affiliations:** ^1^ Department of Parasitology Medical Faculty, Center for Infectious Diseases Heidelberg University Heidelberg Germany; ^2^ Institute of Theoretical Physics and BioQuant Heidelberg University Heidelberg Germany; ^3^ German Center for Infection Research Partner Site Heidelberg Heidelberg Germany

**Keywords:** cell migration, gliding motility, *Plasmodium*, traction force microscopy

## Abstract

*Plasmodium* sporozoites are the highly polarised and motile forms of the malaria parasite transmitted by mosquitoes to the vertebrate hosts. Sporozoites use myosin motors to generate retrograde flow of actin filaments. These are linked to plasma membrane spanning adhesins, which in turn bind to the extracellular environment, resulting in forward directed gliding motility. The gliding motility machine of sporozoites leads to high speeds in the range of micrometres per second, which are essential for efficient migration in the skin. Yet, it is not clear how the individual parts of the machinery work together to generate force during migration. Sporozoites are elongated and curved cells and move on circular tracks in vitro. Sporozoites lacking the adhesin thrombospondin‐related anonymous protein (TRAP) like protein, TLP, can still migrate in the skin, but at a lower level. TLP lacking sporozoites generate a lower force on the dorsal (nonsubstrate facing) surface as measured by laser tweezers. Here we use traction force microscopy to investigate motile sporozoites and the forces they produce during migration on their ventral surface. Both wild type and *tlp(‐)* sporozoites show distinct foci of force generation, but *tlp(‐)* sporozoites generating overall lower forces. Our findings demonstrate that TLP is an important element of the force‐generating machinery during sporozoite gliding motility.

## 1 INTRODUCTION

**FIGURE 1:**
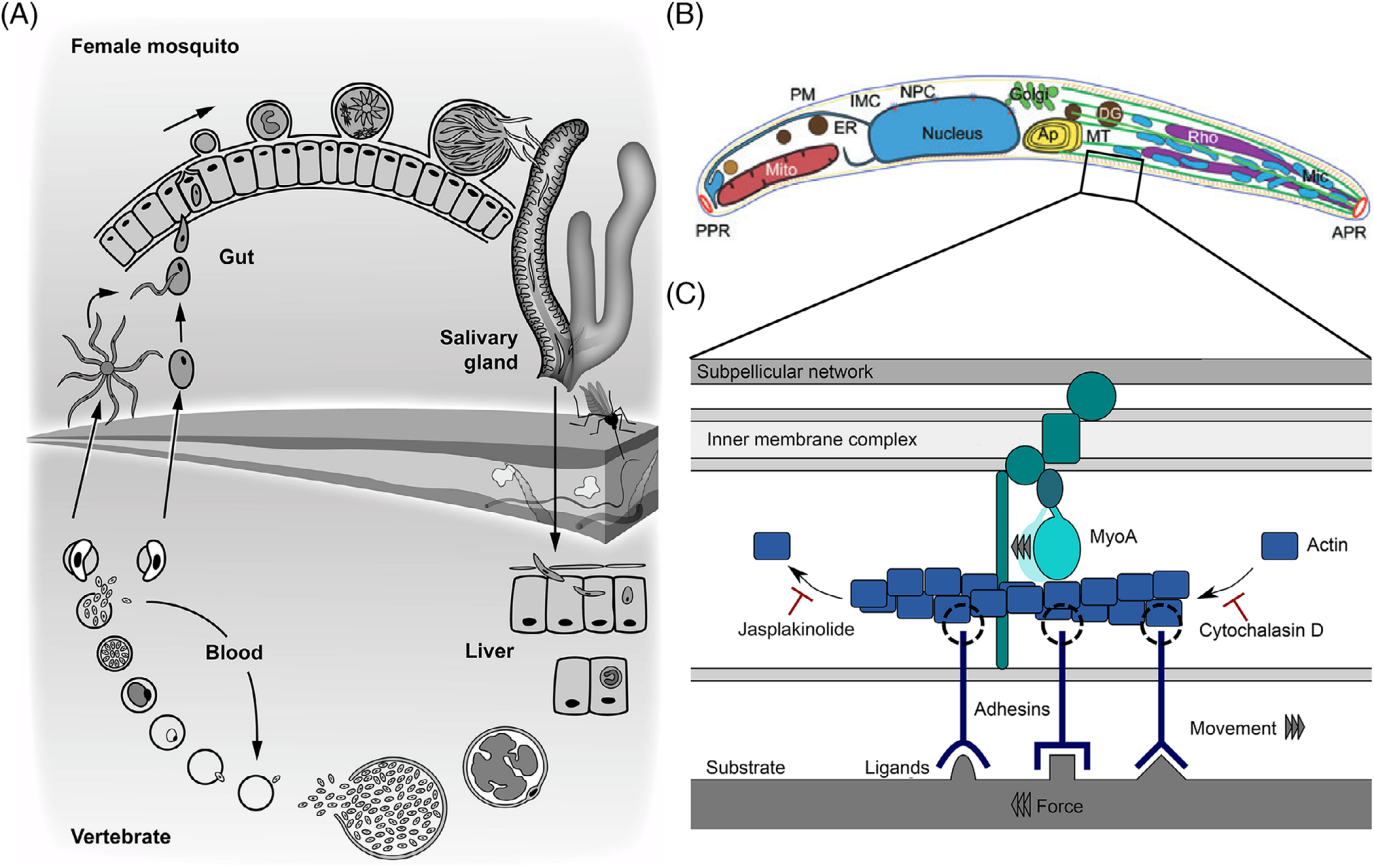
*Plasmodium* parasites alternate between intracellular growth and extracellular motility. (A) Life cycle of *Plasmodium*. *Plasmodium* parasites replicate in the erythrocytes of the vertebrate host and are taken up with the blood meal by a female *Anopheles* mosquito. Mating occurs inside the midgut and a motile ookinete traverses the midgut epithelia and transforms into an oocyst where thousands of sporozoites are formed. They egress and invade salivary glands, where they mature before they are injected into the skin of the vertebrate host. Sporozoites actively leave the skin, invade a blood vessel and once in the liver, invade hepatocytes where they mature into blood infective forms, closing the life cycle. (B) The motor machinery is located below the plasma membrane of the sporozoite. The subpellicular network is tightly interwoven with the inner membrane complex, where the motor, myosin A is anchored. Actin filaments are polymerised at the apical end of the parasite by Formin 1 (not depicted) and moved towards the basal end via myosin A. Plasma membrane spanning adhesins bound to extracellular ligands are moved along with actin filaments, generating a rearwards directed force, resulting in forward movement.

Malaria is transmitted during the bite of a mosquito, when the insect injects *Plasmodium* sporozoites into the skin of its vertebrate host. These sporozoites are formed in oocysts on the mosquito midgut, accumulate in the salivary gland of the insect, are injected during a blood meal and differentiate in the liver of mammals into red blood cell infecting forms that cause the disease (Figure 1A). Sporozoites leave oocysts in an active process, then migrate to enter and disperse within salivary glands, cross the dermis of the skin to enter into blood vessels and actively exit the blood stream in the liver to infect hepatocytes.^1^, ^2^, ^3^ To achieve these tasks the parasite employs gliding motility, a form of substrate‐based locomotion that is very fast because it does not require any cell shape changes.^4^, ^5^ Sporozoites isolated from the oocysts are only weakly migratory, while those isolated from the salivary glands are highly motile.^6^ Also, within salivary glands, sporozoites are slow, but once injected into the dermis, they migrate rapidly. This suggests that motility can be modulated by external cues that are translated into signaling events.^7^, ^8^ The journey of sporozoites provides a formidable challenge for microscopists to image.^9^ Early electron micrographs revealed the cellular processes during sporozoite formation in the oocysts,^10^ ^‐–^ ^12^ cryo‐electron tomography revealed structural details of, for example, microtubules,^13^, ^14^, ^15^ while in vivo imaging revealed sporozoite migration in the skin and liver.^16^, ^17^, ^18^, ^19^, ^20^ Isolated sporozoites are crescent‐shaped cells that move with their apical (front) end leading.^2^ In addition to being curved, they are also chiral. On a flat support, sporozoites move in a circular manner mainly in counterclockwise direction^21^ and in 3D isotropic environments, they move with near perfect helical trajectories, which appear to be right‐handed.^22^, ^23^ Surface sensitive imaging modalities such as total internal reflection microscopy, reflection interference contrast microscopy and traction force microscopy have shown that sporozoites form distinct adhesion sites on a substrate that are turned over rapidly during migration.^24^, ^25^

Sporozoites, like other motile forms of *Plasmodium* or related parasites, power their motility by an actin–myosin driven motor machinery that resides underneath the plasma membrane (Figure 1B, C). It appears to be linked via the C‐terminus of transmembrane adhesins to the substrate on which the sporozoites move.^4^, ^5^ Adhesins are secreted at the front end of the parasites and transported rearwards by myosin. Such a retrograde flow can be observed for actin filaments by single molecule microscopy^26^ or for adhesins, when mosquito debris or polystyrene beads attach to the parasite.^27^ Different adhesins appear to fulfil different roles on sporozoites. The thrombospondin‐related protein 1 (TRP1) is essential for the onset of sporozoite motility in oocysts and hence egress of sporozoites.^28^ TRP1 is further essential for entry into salivary glands. The thrombospondin‐related anonymous protein (TRAP) is not required for egress from oocysts but essential for entry into salivary glands, migration in the dermis and entry into hepatocytes.^29^ The TRAP‐related protein (TREP, initially named S6 or UOS3) is important for salivary gland entry but not for hepatocyte invasion.^30^, ^31^, ^32^, ^33^ The TRAP‐like protein (TLP) is dispensable for salivary gland entry, but plays a role in migration within the dermis.^34^, ^35^, ^36^

Laser tweezers have been used to investigate the roles of TRAP and TREP in parasites isolated from the mosquito circulatory fluid, the haemolymph.^24^ This revealed differential roles of the two proteins in distribution and force generation on the surface. The role of TLP has been investigated in the highly motile sporozoites isolated from the salivary glands. Sporozoites lacking TLP revealed a lower capacity for force generation and a faster retrograde flow.^26^ Furthermore, these *tlp(‐)* sporozoites also migrated less robustly on soft substrates than did wild type parasites.^37^ Intriguingly, adding a low dose of the actin depolymerisation inhibitor jasplakinolide could partially rescue this deficiency, suggesting a direct link between TLP and actin filaments.^37^

Sporozoites exhibit chiral motion patterns and this chirality is most likely caused by the cytoskeleton,^38^ as has recently been shown for the related apicomplexan parasite *Toxoplasma gondii*.^39^ In particular, the asymmetric microtubule corset anchored in the apical ring, might generate the curved shape^40^ and a dorsoventral polarity.^15^ This polarity might influence the forces generated on the ventral side as can be measured by traction force microscopy^25^ and those on the dorsal side as measured by beads held in an optical trap^26^ and therefore limit the significance of investigating the latter to learn about the former. To investigate how forces on the different surfaces correlate and which roles are played by the different molecular players, we here probed the capacity of both wild type and *tlp(‐)* sporozoites to generate traction forces on their ventral side.

## 2 RESULTS

**FIGURE 2:**
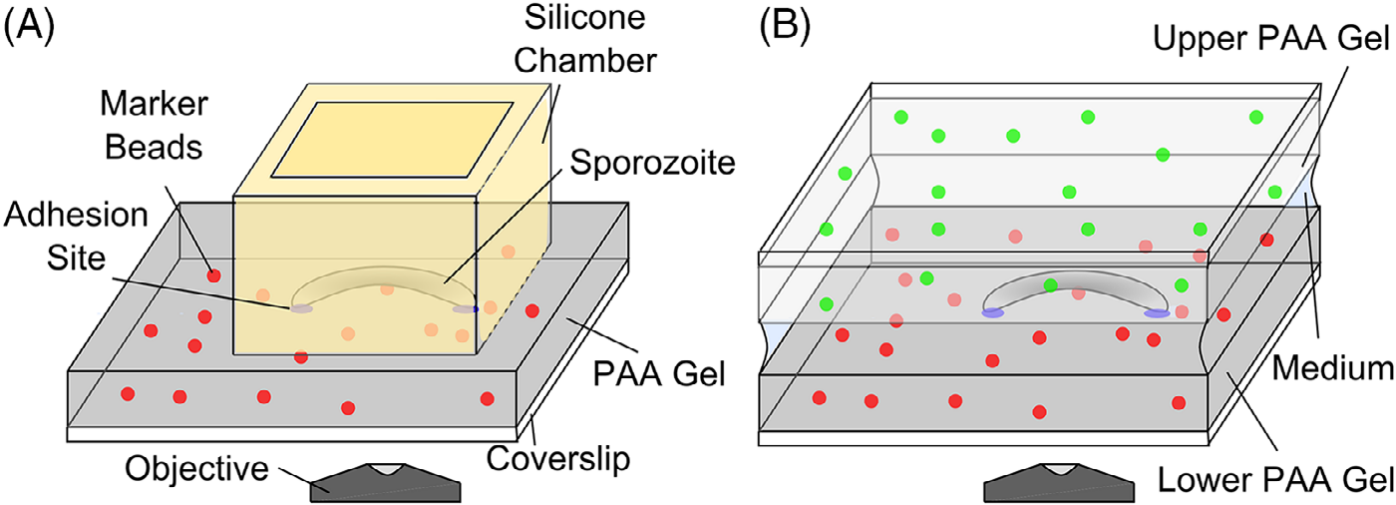
Setups for parasite gliding assays and traction force microscopy. (A) A silicone chamber (yellow) was placed on top of an PAA gel (grey) with red marker beads embedded. The sporozoite solution was pipetted into the chamber. Imaging was performed through the hydrogel using an inverted spinning disc confocal microscope. This 1‐gel system was used for experiments presented in Figure 3. (B) For all TFM experiments, sporozoites were sandwiched between two PAA gels, the lower one containing red and the upper gel green marker beads. The distance between the gels was at least 20 µm, such that sporozoites were only in contact with one of the gels. Imaging thus resolved only the beads on the lower PAA gel. Beads in the upper gel were only used for orientation.

**FIGURE 3:**
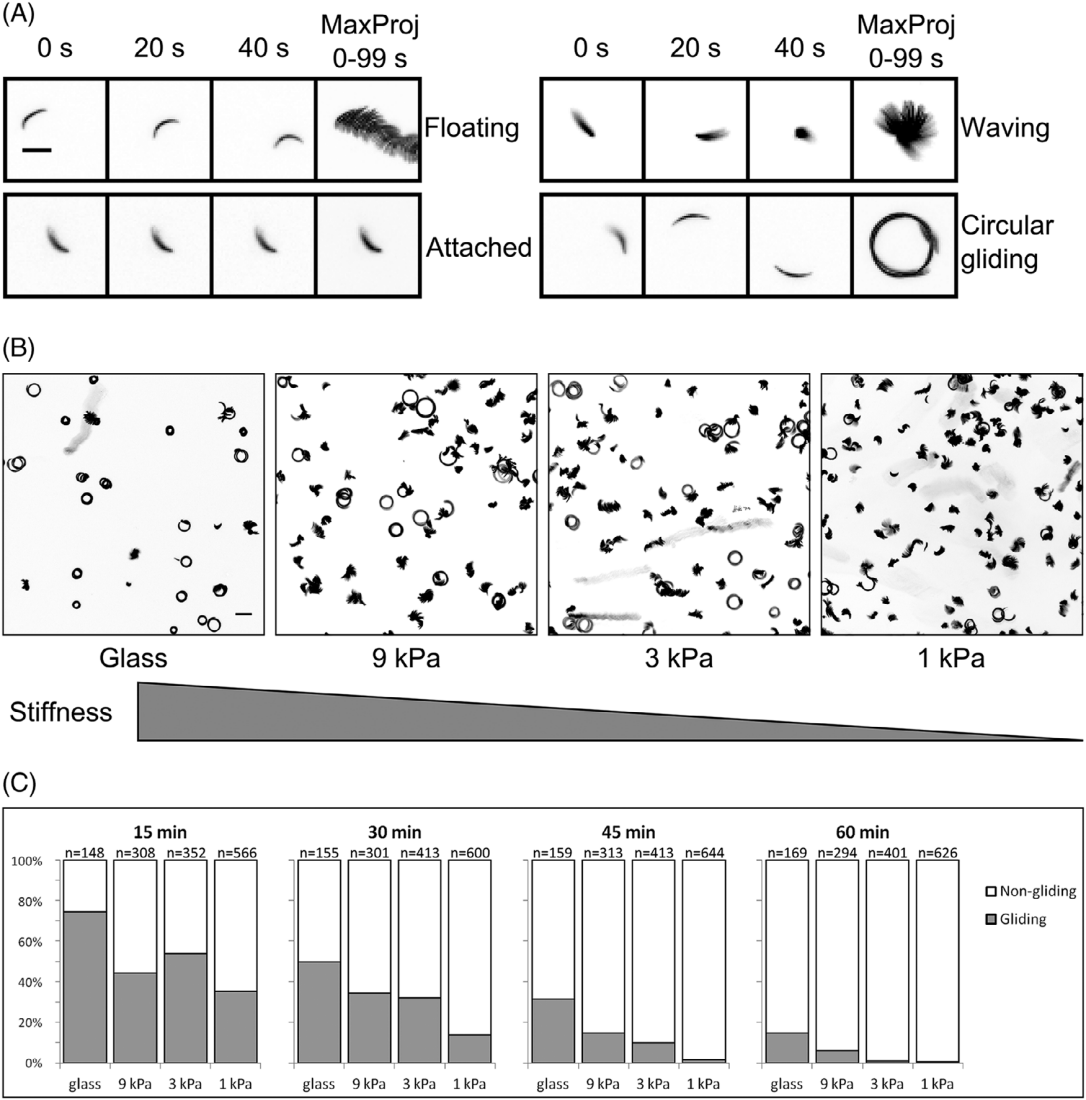
Sporozoite move best on less elastic substrates. (A) Motility patterns of sporozoites on elastic substrates (1‐gel system). Fluorescence images of sporozoites at indicated time points and maximum intensity projections. Sporozoites are either not attached and float in the medium, attached without moving, attached at only one side (waving) or moving on circular trajectories. Scale bar, 10 µm. (B) Maximum intensity projections (0–99 s) of sporozoites on substrates of different elasticity as indicated 30 min after activation. Graph not to scale. Scale bar, 20 µm. (C) Quantitative analysis of sporozoites gliding on different elastic substrates as shown in (B) at indicated time points after sporozoite activation. All sporozoites moving for at least half a circle within 100 s of recording were defined as gliding. Numbers of investigated sporozoites are shown on the top of each bar. Fisher's exact test was used to calculate differences between 9 and 1 kPa gels and revealed *p* < 0.02 for 15 min and *p* < 0.001 for at 30, 45 and 60 min.

**FIGURE 4:**
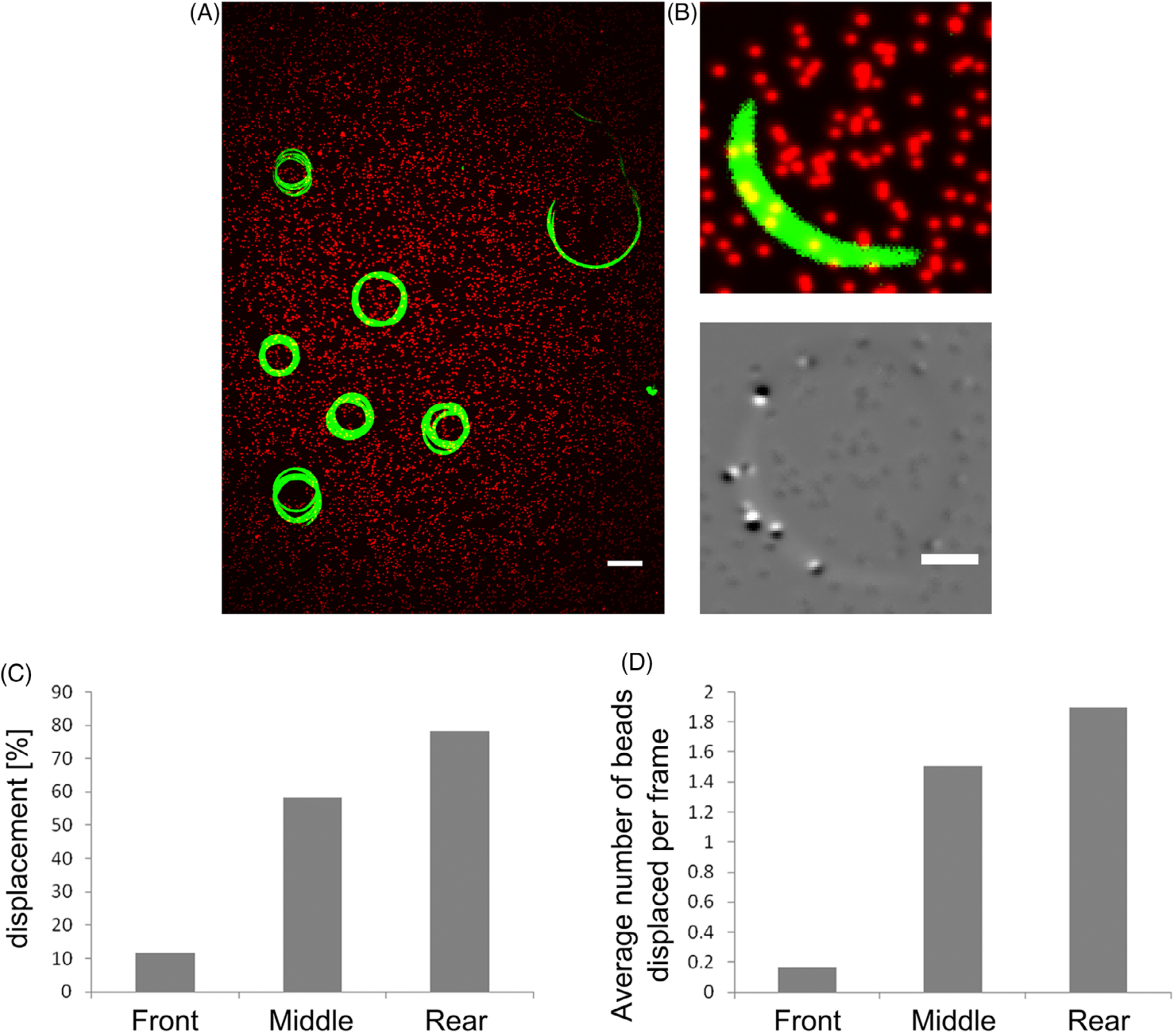
Traction force microscopy of sporozoites. (A) Maximum intensity projection (0–59 s) of 7 sporozoites (green) gliding on a PAA gel that contains red fluorescent marker beads. Shown is one field of view as observed with an inverted microscope using a 60× objective. Scale bar, 10 µm. (B) Top: Snapshot of a single sporozoite on an elastic gel with embedded marker beads. Bottom: Overlay of beads in relaxed (black) and in displaced (white) position as the sporozoite moves over the gel. Scale bar, 2 µm. (C) Top: Percentage of frames with bead displacement within the indicated subcellular regions (*n* = 11 sporozoites for each group, 25 frames per sporozoite, see Excel file containing raw data in Supplementary Data). These numbers imply that forces were above the background level at this fraction of time points. Bottom: Average numbers of beads displaced per frame within the indicated subcellular regions of those sporozoites shown above. Higher numbers indicate a larger area of gel deformation. Statistical analysis using a Kruskal–Wallis test revealed *p* < 0.0001 between the individual bars.

To isolate sporozoites we infected *Anopheles stephensi* mosquitoes either with wild type‐like (wt) *Plasmodium berghei* (strain ANKA) parasites that expressed the green fluorescent protein (GFP) in their cytosol under the control of the ef1α promoter and mCherry under the sporozoite specific *csp* promoter^28^ or with *tlp(‐)* parasites lacking the gene encoding TLP and expressing GFP under the control of the ef1α promoter.^35^ 17–20 days after infection, salivary glands of these mosquitoes were extracted and sporozoites isolated. Sporozoites glide on glass with average speeds of 1–2 µm/s on predominantly circular paths, but also show other movement patterns.^24^, ^25^, ^41^ Traction force microscopy (TFM) is often performed by placing cells on polyacrylamide (PAA) gels coated with adhesive substances and containing fluorescent beads (TF gels).^42^, ^43^ We found previously that sporozoites also move on noncoated TF gels, probably as proteins such as serum albumin of the medium adsorb to the substrate.^25^ We employed two set‐ups to first evaluate sporozoite migration on soft gels, whereby a silicon chamber was put on a TF gel (Figure 2A, employed to measure differences in overall sporozoite motility) and secondly a sandwich assay where sporozoites were placed between two TF gels that are about 20 µm apart (Figure 2B, employed for all traction force microscopy). In both cases the PAA gels were spiked with red fluorescent 200 nm marker beads, while in the second case the upper gel of the sandwich was spiked with green fluorescent beads. While on glass most sporozoites do move in circles, others also float or stay attached to the substrate without movement or attach with one end to the substrate and move their bodies, also in a circular fashion, a movement termed waving^21^ (Figure 3A). We placed sporozoites on either glass or three types of TF gels that differed in their stiffness, and imaged the parasites at different times post placement. This revealed that sporozoites move most robustly on stiffer substrates (Figure 3B and C), confirming our previous data.^23^ Also, the average speed of motile sporozoites followed the same trend (Figure S1). We next investigated sporozoites moving in the sandwich setup by spinning disc confocal microscopy using a 60× 1.2 NA air objective to image bead displacements. To this end we placed between 50,000 and 100,000 sporozoites on a gel allowing to image several sporozoites as they migrated (Figure 4A). We next measured bead displacements using FIJI^44^ to get a rough estimate of traction forces. This revealed that beads were displaced by a maximum of 0.4 µm (Figure 4B). Next, we measured the average number of beads displaced per frame within different subcellular regions of 11 sporozoites imaged for 25 frames at 1 frame per second. These regions were simply defined as the front, middle and last third of the sporozoite. As bead density varies in the gels, we only compared sporozoites moving on similarly dense bead patterns. This analysis showed that more beads were displaced in the central (middle) and rear regions compared to the front (Figure 4C and D).

To quantify traction forces, we used our custom‐written software.^45^ Bead displacements was calculated using particle image velocimetry (PIV) with a window size of 32 pixels. Average intensity projections of 30 consecutive frames served as reference image to measure bead displacements against. Drift correction was carried out using the lower right corner of each image, where no bead displacements were observed due to the large distance to the sporozoites. We discarded sporozoites where beads did not relax back to their initial state or where the focus shifted during imaging. Traction forces were then determined from displacement vectors using established Fourier transform traction cytometry (FTTC) methods^46^ (Figure S2). Because resolution was not sufficient to reconstruct all forces within the contour of the sporozoites, we introduced an effective cell contour that was 25 pixels (around 2.5 µm) larger than the sporozoite contour (Figure S2, panel 4). To analyse traction forces within certain regions of the cell, a polygonal region of interest (ROI) was manually selected and peak and mean traction forces within this ROI were automatically determined with the python module ‘roipoly’.

**FIGURE 5:**
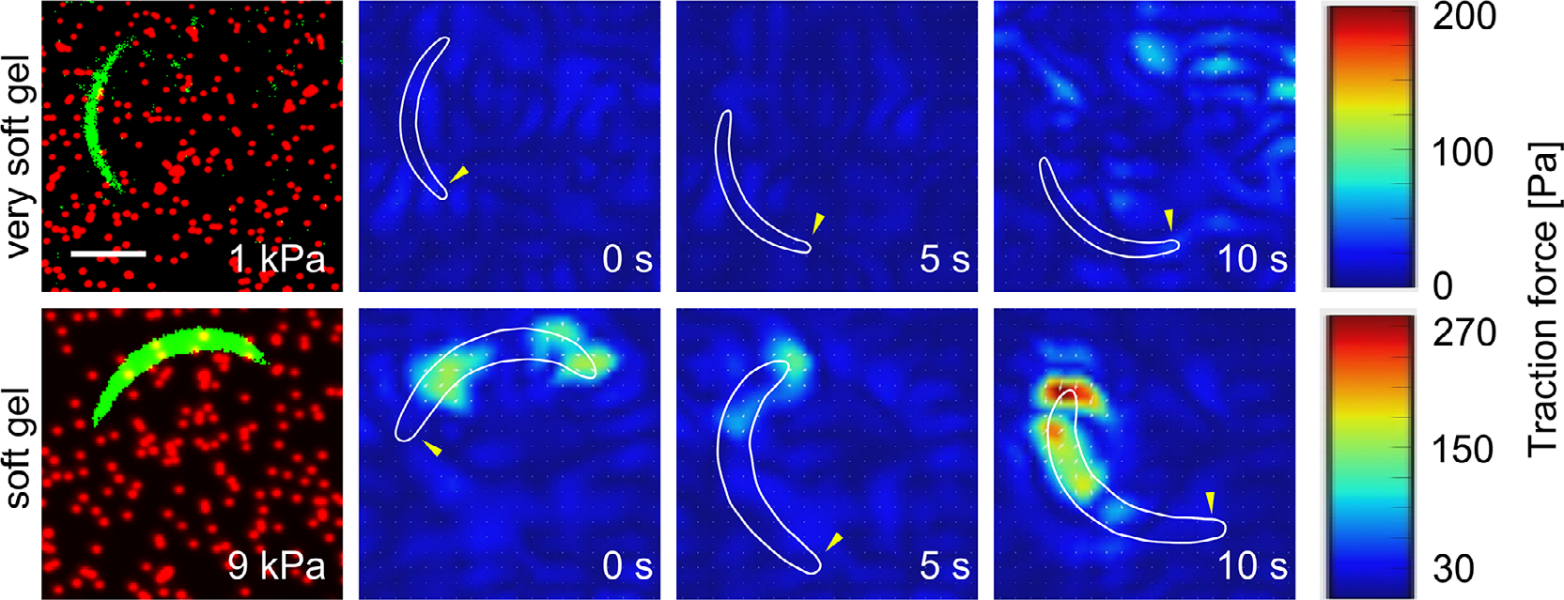
Traction forces depend on gel elasticity. Example images of GFP‐expressing sporozoites (green) on flexible substrates of different stiffness containing fluorescent red marker beads followed by traction forces of migrating sporozoites at multiple time points. The cell contour is depicted in white. Yellow arrowheads indicate the apical tip of the sporozoite. Scale bar, 5 µm.

We first imaged sporozoites on the softest (1 kPa) and stiffest (9 kPa) TF gels. Sporozoites migrating on 1 kPa gels did not displace beads and hence no traction force could be calculated. In contrast, beads were readily displaced on 9 kPa gels revealing traction stresses of up to 250 Pa (Figure 5). We thus continued to only work with the 9 kPa gels and compared on those the motility of wt and *tlp(‐)* sporozoites.

**FIGURE 6:**
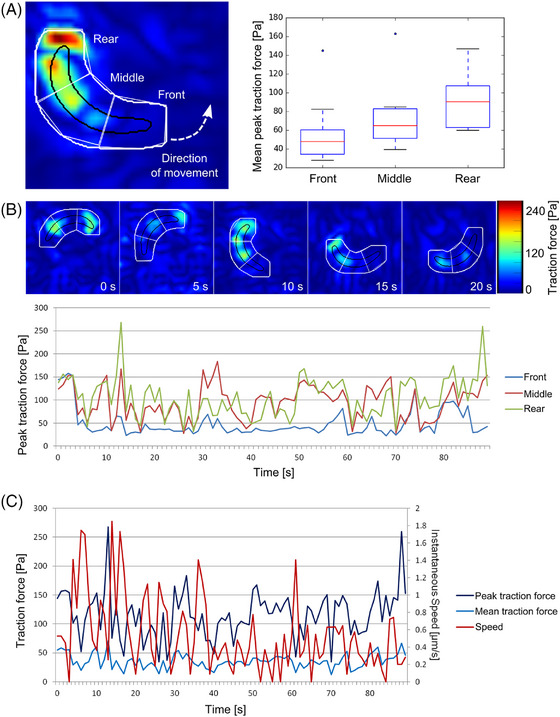
Spatiotemporal dynamics of traction forces. (A) Example image and quantified traction forces at front, middle and rear of 10 gliding sporozoites imaged on three different days (30 frames analysed per sporozoite). Kruskal–Wallis test: *p* < 0.02. (B) Heat maps and plot showing traction forces over time for a sporozoite. The cell contour is indicated in black. To include deformations at the rim of the sporozoite as well, the cell contour was enlarged by 2.5 µm in every direction (depicted in white). (C) Traction forces and instantaneous speed over time for the same sporozoite as shown in (B) imaged at 1 Hz.

**FIGURE 7:**
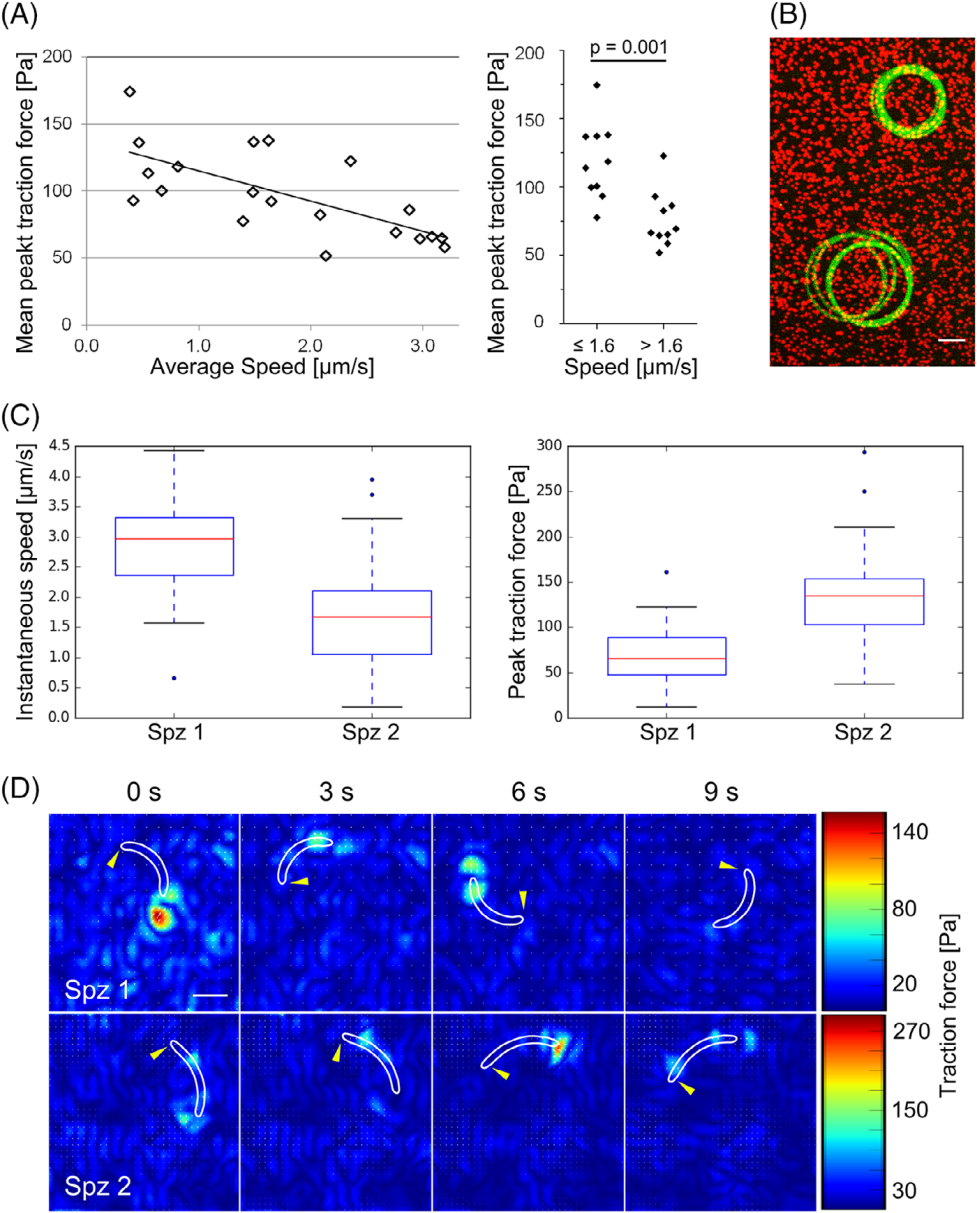
Correlation of traction force and speed. (A) Plot of mean peak traction force versus average speed of 20 sporozoites (30 frames per sporozoite). Force transmission negatively correlates with speed (*R* ^2^ = 0.48). Right: Traction forces of the 10 fastest sporozoites (> 1.6 µm/s) are significantly (*p* < 0.001; unpaired *t*‐test) lower than traction forces of the 10 slowest sporozoites (≤ 1.6 µm/s). Data from imaging experiments performed on two different days. (B) Maximum intensity projection of a fast and a slow sporozoite depicted in green gliding next to each other on a PAA gel containing red fluorescent marker beads. Scale bar, 10 µm. (C) Quantitative analysis of instantaneous speed and traction force of the fast sporozoite (spz 1) and the slow sporozoite (spz 2) from 12 frames each. Nonparametric Mann–Witney test for both speed and force: *p* < 0.002. (D) Heat maps showing traction forces of the fast and the slow sporozoite. Cell contours are depicted in white. Yellow arrowheads indicate apical tips of the sporozoites. Scale bar, 5 µm.

**FIGURE 8:**
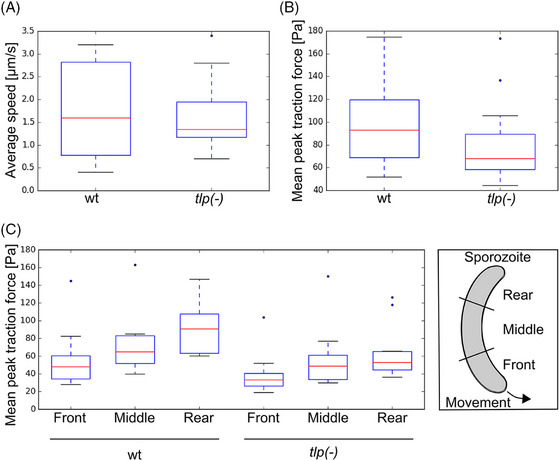
TLP modulates force transmission. (A) Speed of wild type (wt) and *tlp(‐)* mutant sporozoites that were analysed for force transmission (*n* = 20 sporozoites for each group from 3 different experiments at three different days, 30 frames per sporozoite; *p* = 0.64 as determined by nonparametric Mann–Witney test). (B) Traction forces of the wt and *tlp(‐)* mutant sporozoites shown in (A). Parasites lacking TLP generate significantly less traction force (*p* < 0.05) as determined by a nonparametric Mann–Whitney test. (C) Traction forces of wt and *tlp(‐)* mutant sporozoites at front, middle and rear as defined in the scheme (*n* = 10 sporozoites for each group, 30 frames per sporozoite). Only the difference at the rear is significant (*p* < 0.05; multiple parameter Mann–Witney *U* test).

As shown before,^25^ traction forces were largest at the rear of the sporozoite and weakest at the front (Figure 6A and B). Plotting the peak and mean traction force together with the speed of the sporozoites showed that a decrease of peak force was followed by speed peaks, indicating some kind of sporozoite–substrate rupture event (Figure 6C). We imaged 20 wt sporozo their traction forces. Plotting the mean peak traction force of the individual parasites against their average speed revealed an inverted correlation (Figure 7A). Investigating two wt parasites moving next to each other with different speeds showed that the peak traction force was indeed higher in the slow moving sporozoite, suggesting that strong adhesion impedes motion (Figure 7B and C). For comparison of wt with *tlp(‐)* sporozoites we thus specifically selected 20 parasites of each line that moved at similar speeds (Figure 8A). This revealed lower mean peak traction forces for *tlp(‐)* compared to wild type sporozoites (Figure 8B). A lower traction force was indeed detected at the front, middle and rear of the *tlp(‐)* sporozoites (Figure 8C). Taken together, these data show a decreased traction force on the ventral side of *tlp(‐)* sporozoites, which is in line with force measurements on the dorsal side using laser tweezers and polystyrene beads.^26^

## 3 DISCUSSION

Traction force microscopy of motile *Plasmodium* sporozoites had revealed for the first time that adhesion to the substrate impedes motion and that sporozoites have to rupture bonds at their rear in order to move forward.^25^ These findings agree with the fact that the cleavage of adhesins by the rhomboid protease is required for fast gliding motility.^47^, ^48^ Compared with the first study on traction forces of sporozoites,^25^ here we did not use a mix of different methods, but relied on the well‐established framework of regularised Fourier transform traction cytometry (FTTC),^45^, ^46^ adapted to the small size of the sporozoites by introducing an enlarged footprint area. Because the sporozoite is a long and slender cell, this procedure preserved the front‐back polarity which is essential for this study. Overall, our improved procedures enable higher throughput data analysis. We used the method to compare wild type sporozoites with a transgenic parasite line lacking the surface protein TLP and found that these sporozoites produce less traction force during gliding motility.

Sporozoites move at remarkably high speed in the skin, outpacing neutrophils by one order of magnitude. How the different types of adhesins on the surface of the parasite play together to enable this motility is not known. At least four transmembrane proteins play a role in gliding, TRAP, TREP, TLP and TRP1.^28^ ^–‐^ ^36^ Deletion of TRP1 leads to a complete block in oocyst egress. Deletion of TRAP and TREP leads to a complete and partial block in salivary gland invasion, respectively. Deletion of TLP shows the least prominent phenotypic differences to the wild type in mosquitoes, as expression of TLP only starts once sporozoites are inside salivary glands.^36^ As parasites mature within the glands and only matured sporozoites are able to glide robustly, we are currently able to only investigate *tlp(‐)* sporozoites with advanced imaging methods.

The use of high‐speed imaging coupled with optical tweezers trapping beads on the sporozoite surface has revealed that *tlp(‐)* sporozoites show a faster retrograde membrane flow, but produced less force than wild type sporozoites.^26^ This suggests that TLP is needed to somehow organise force generation. This hypothesis was strengthened by experiments using laser traps and beads on wild type sporozoites treated with the actin stabilising drug jasplakinolide. These showed less force and higher retrograde flow rate, similar to *tlp(‐)* sporozoites.^26^ Similarly, using traction force microscopy with actin modulating drugs, wild type parasites were found to produce less force upon addition of low concentrations of either actin filament depolymerising (cytochalasin D) or stabilising (jasplakinolide) drugs.^25^ Together these experiments suggest that fine‐tuned actin filament organisation is needed for optimal force generation. The experiments presented in our work here further strengthen these findings and provide evidence that gene deletion mutants have similar effects on force generation on the dorsal and ventral sides of the chiral sporozoite. Direct comparison of absolute force generation at the dorsal and ventral side with the same method would be interesting to test these assumptions.

The technical improvements presented here compared to our initial TFM presented in Münter et al.^25^ also suggest that it should be possible to image motile parasites derived from the haemolymph of the mosquitoes. Of those only about 10% show robust gliding, compared to over 60% in salivary gland‐derived sporozoites. While still cumbersome, imaging haemolymph‐derived sporozoites would allow the investigation by TFM of a much larger set of mutant parasites with defects in gliding motility, for example, parasites lacking different domains of TRAP or TRP1 or with discrete point mutations in actin and myosin. Their investigation could reveal new insights into how the different adhesion sites along the sporozoite play together to move parasites forward at these high speeds.

In conclusion, we showed here that sporozoites lacking *tlp(‐)* exert less force on their ventral sides, corresponding to the lower force generation measured before on the dorsal side by laser tweezers. This shows that among the different surface adhesins, although being not essential, TLP plays a role in modulating gliding motility.

## 4 MATERIALS AND METHODS

### 4.1 Animals and infection

The rodent infecting *Plasmodium berghei* was used to produce sporozoites. Briefly, after infection of mice with blood stage parasites, 3‐ to 7‐day‐old *Anopheles stephensi* mosquitoes were allowed to feed for 20 min on anaesthetised (ketamin and xylazine (100 mg/kg weight and 10 mg/kg weight, respectively)) mice once motile microgametes could be observed in a drop of blood under an Axiostar plus transmitted‐light microscope (Zeiss). Mosquitoes were kept in incubators at 21°C and > 70% humidity. 17 to 25 days after mosquito infection, the sporozoites were isolated from the salivary glands of 5–10 infected mosquitoes as determined by the fluorescent signal from oocysts in the midgut. To this end, the salivary glands were dissected under a Nikon SMZ1500 microscope with a fluorescence unit attached and immediately put into phosphate buffered saline (PBS) on ice. Shortly before the experiments salivary glands were gently homogenised using a plastic pestle. The mixture was centrifuged at 7000 rpm in a Biofuge pico microcentrifuge (Heraeus) for 2 min. The pellet containing the salivary gland sporozoites was resuspended in RPMI medium supplemented with 1× penicillin/streptomycin and 3% bovine serum albumin (BSA) to activate the sporozoites.^21^, ^49^ We used multiple sporozoite preparations from different sets of mosquitoes for the analysis as we prepared them fresh before each imaging session.

### 4.2 Preparation of elastic polyacrylamide (PAA) substrates

Covalent attachment of thin PAA hydrogels to a glass surface requires its chemical activation. Thus, 24 × 40 mm glass coverslips were silanised similar to Ref. (50). To this end, coverslips were passed through the flame of a Bunsen burner. Subsequently, a drop of 0.1 M NaOH was spread onto the glass surface using the side of a Pasteur pipette. After drying on air, the glass surface was covered with 3‐Aminopropyltrimethoxysilane (APTMS) for 4–5 min and washed extensively with distilled water. Then, they were incubated in PBS containing 0.5% glutaraldehyde for 30 min. The coverslips were washed extensively with multiple changes of distilled water and allowed to dry on air. Functionalised glass coverslips were stored at room temperature for up to one month. The elasticity of PAA gels was adjusted by varying the relative concentration of the monomer and the cross‐linker.^50^ PAA gels were prepared with elastic moduli ranging from about 1 to 9 kPa according to Ref. (51). To this end, AA and Bis‐acrylamide were mixed with PBS to obtain a final concentration of 5% AA and 0.03%, 0.1% or 0.3% Bis‐acrylamide. Note that the published values for the elastic modulus were obtained for gels that were polymerised in water and might therefore be slightly lower. The gels were polymerised in PBS to prevent them from swelling when transferred into cell culture medium. A stock solution of AA/Bis‐acrylamide was prepared to reduce differences in gel elasticity among different experiments. 200 nm red fluorescent marker beads (excitation 580 nm/emission 605 nm) were diluted 1:200 in the prepolymer solution. Dissolved oxygen was removed by degassing for at least 30 min within a desiccator. Polymerisation was started in a wet chamber to avoid drying by adding APS and TEMED to the prepolymer solution shortly before pipetting 30 µL onto a nontreated 22 × 22 mm glass coverslip. The drop was immediately covered with a functionalised glass coverslip. During polymerisation the beads moved towards the bottom coverslip, possibly by gravity. The gel was transferred after 15 min into PBS and the nonfunctionalised coverslip was removed carefully after another 15 min. Marker beads were mostly located within one plane at the gel surface as seen by imaging. The gel thickness was about 60 µm as estimated by the volume of prepolymer solution and the area of the gel dictated by the glass coverslip. This thickness was chosen for the gel to be thick enough that forces applied by the parasites to the gel surface are not influenced by the glass coverslip^52^ but thin enough to allow for imaging through the gel. The hydrogels were kept in PBS at 4°C and stored for a maximum of three days.

### 4.3 Gliding assays

PAA gels were incubated in RPMI medium containing 3% BSA for 10 min. A silicone flexiPERM chamber (Greiner BioOne) was put on the PAA gel to prevent flow and restrict the sporozoites to a smaller area and thereby increase the cell density on the elastic substrate (Figure 2A). Subsequently, 15 µL of sporozoite solution were pipetted into the middle of the well. The sporozoites were allowed to settle for 7 min. Then, the chamber was filled up with 80 µL of RPMI medium containing 3% BSA to prevent the gel from drying out during imaging. Alternatively, a 24 × 40 mm glass coverslip cleaned with acetone and isopropanol was used as substrate. Sporozoites expressing cytosolic green fluorescent protein (GFP) under the sporozoite stage‐specific circumsporozoite protein (CSP) promoter^53^ were used. Images were collected with an inverted widefield fluorescent Axiovert 200 M microscope (Zeiss) using a 10× objective (NA 0.5) and Axiovision 4.8 software. Images were recorded 15, 30, 45 and 60 min after activation of the sporozoites for 100 s at 1 Hertz (Hz). Imaging was performed at room temperature. These assays were performed once per gel and time point using high numbers of sporozoites with the positive control (glass) performed last.

### 4.4 Traction force microscopy (TFM)

TFM was performed using sporozoites expressing GFP under the control of the ef1α promoter and mCherry under the CS promoter^7^ as well as with *tlp(‐)* sporozoites expressing GFP under the control of the ef1α promoter.^35^ Shortly before imaging, PAA gels were incubated in RPMI medium containing 3% BSA. 15 µL of sporozoite solution were pipetted onto the lower PAA gel. To prevent the solution from evaporating during imaging, the sporozoite solution was covered with a second PAA gel (Figure 2B). The resulting gap between the two gels was measured by focusing the microscope from the surface of the lower gel to the surface of the upper gel and measuring the displacement of the objective lens. The distance between the two gels was between 20 and 70 µm. Hence, sporozoites with a thickness of about 1 µm only form adhesions to one gel. TFM was performed at room temperature and started about 15 min after activation of sporozoites and the experiments were terminated after a maximum of 1 h. Imaging was performed at 1 Hz using an UltraView spinning disk confocal unit (Perkin Elmer) on an Eclipse Ti inverted microscope (Nikon) with an ORCA‐Flash4.0 CMOS camera (Hamamatsu, Shizuoka, Japan). Because of the large distance between the glass coverslip and the sporozoites, a 60× water immersion objective (NA 1.2) was used to minimise spherical aberrations. Images were obtained with Volocity 3D Image Analysis Software. Spinning disc microscopy was used to image sporozoites and beads in different channels at high frame rates and to reduce interference resulting from beads that might not be in the focus plane.

### 4.5 Data analysis

Image sequences were imported to Fiji^44^ for analysis. All sporozoites performing at least half a circle per 100 s as seen on maximum intensity projections were defined as gliding. The speed of the parasites was either analysed by counting the number of circles performed within 100 s or by manually tracking the parasites at the front end using the Manual Tracking plugin of Fiji. For traction force analysis, the images were processed according to the workflow shown in Figure S2. Only those sporozoites were analysed, where bead displacement was solely due to traction forces transmitted by the sporozoite, that is, beads were going back to the initial relaxed position after the sporozoite moved over them, no beads appeared or disappeared during imaging and there was no excessive focus‐drift. Traction forces were reconstructed using our custom‐written TFM‐code.^45^ Shortly, bead displacement was calculated using particle image velocimetry (PIV) with a window size of 32 pixels. Average intensity projections of 30 consecutive frames served as reference image. Drift correction was carried out using the lower right corner of each image, where there is no bead displacement as the sporozoite does not move over this site. Traction forces were determined from displacement vectors using FTTC methods.^46^ The resulting force vectors and continuous stress maps were further processed using a python script. Information about the cell contour, received by segmentation, was used to determine peak and mean traction force applied by the sporozoite to the substrate at a certain time point. The resolution of traction force is limited when using the before mentioned method. Therefore, the cell contour was enlarged by 25 pixels corresponding to a distance of about 2.5 µm after segmentation. To analyse traction forces within certain areas of the cell, for instance front, middle and rear, a polygonal ROI was manually selected and peak and mean traction forces within this ROI were automatically determined with the help of the python module roipoly.

### 4.6 Statistical analysis

Box‐and‐whisker plots depict the 25% quantile (blue lower bound), median (red middle line), 75% quantile (blue upper bound) and nearest observations within 1.5 times the interquartile range (whiskers). Outliers beyond this range are shown as blue dots. Graphs were generated with Python or Excel. Statistical analysis was carried out using OriginPro 2016G Software. Grouped data was tested for normal distribution using the Shapiro–Wilk test (alpha level = 0.05). Nonparametric data was tested for significance using a Mann–Whitney test. The unpaired *t*‐test was carried out on parametric data. All figures were generated using Inkscape if not indicated otherwise.

## AUTHOR CONTRIBUTIONS

FF designed the study, JR generated traction force gels performed sporozoite gliding experiments and did traction force analysis, DP developed algorithms and software and helped with traction force analysis, MS provided essential assistance in microscopy, USS and FF supervised the study and acquired funding, FF wrote the initial draft of the manuscript, all authors contributed to the manuscript.

## CONFLICT OF INTEREST STATEMENT

The authors declare no competing financial interest

## Supporting information



Supporting information

Supporting information
